# Supplementary figures and images for: Haploinsufficiency of the schizophrenia and autism risk gene Cyfip1 causes abnormal postnatal hippocampal neurogenesis through microglial and Arp2/3 mediated actin dependent mechanisms
Source: Transl Psychiatry. 2021 May 24;11:313. doi: 10.1038/s41398-021-01415-6 (PMC8144403; doi:10.1038/s41398-021-01415-6)

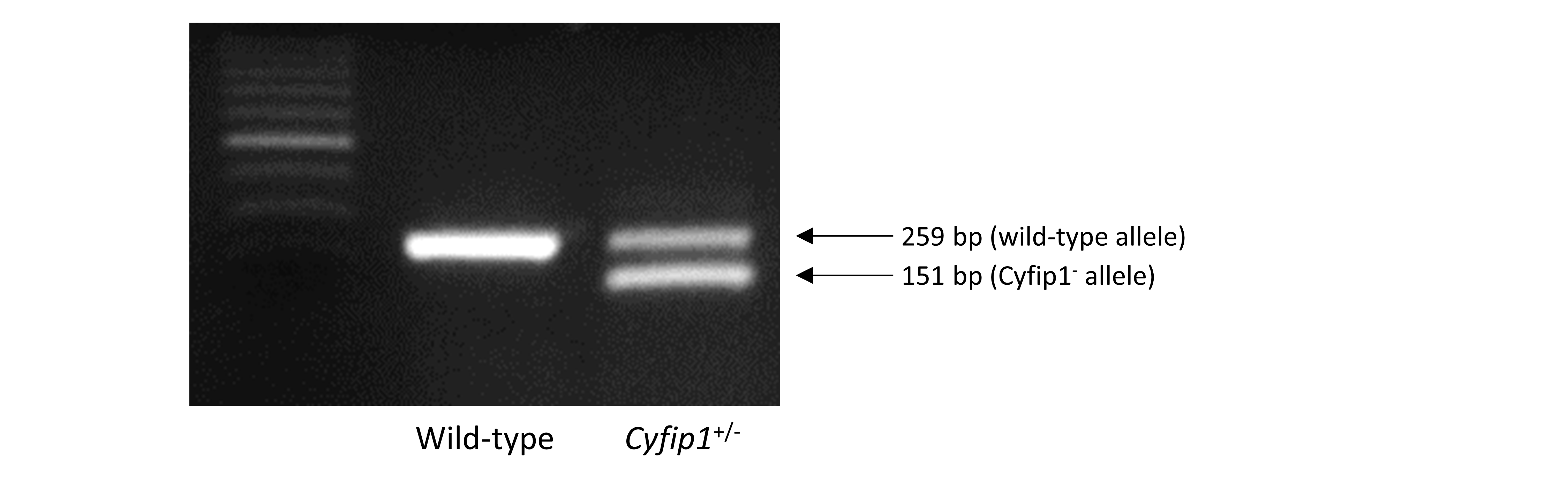

Supplement: Supplementary file 2 — Supplementary figur S1 [file 41398_2021_1415_MOESM2_ESM.tif]

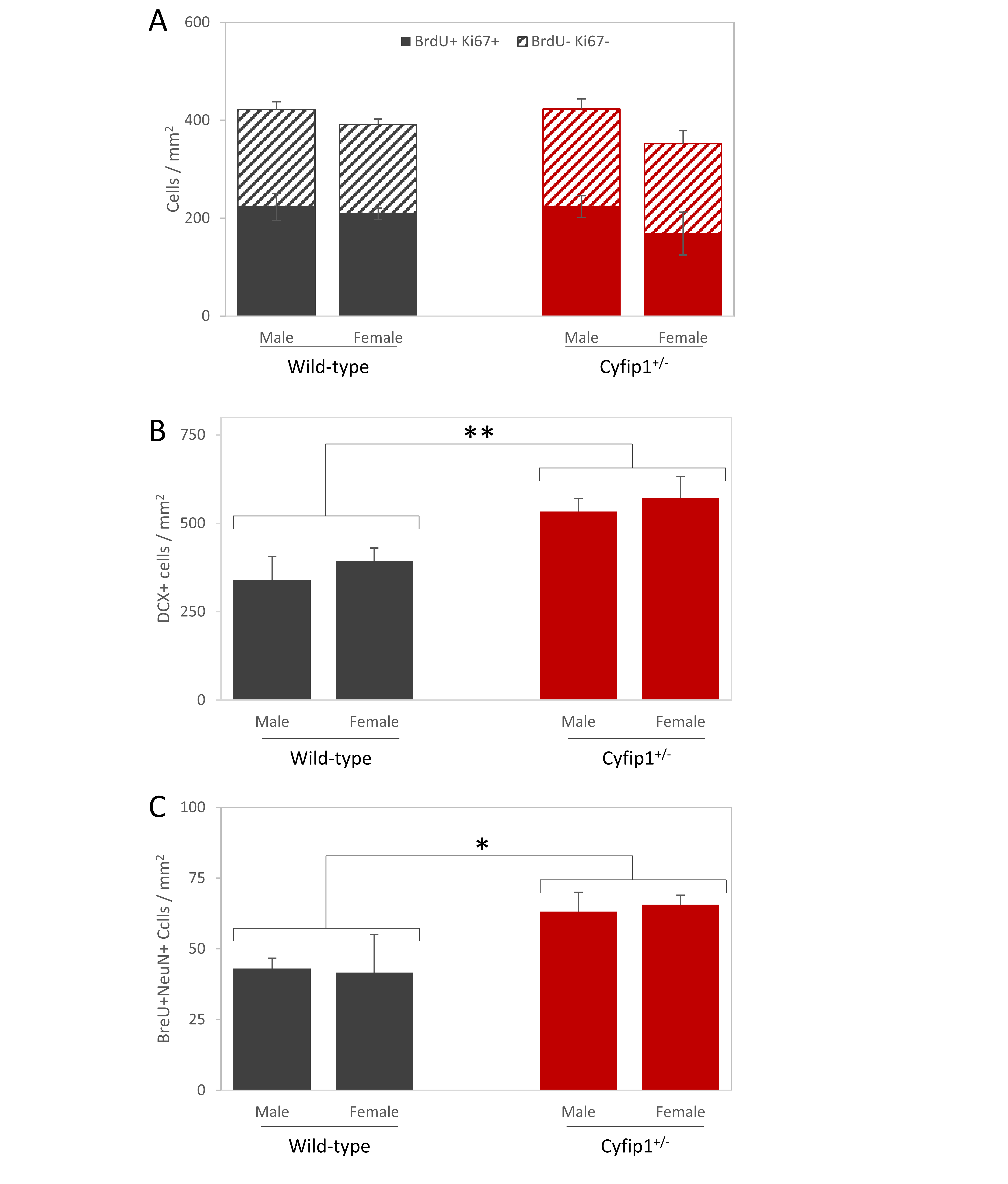

Supplement: Supplementary file 3 — Supplementary figur S2 [file 41398_2021_1415_MOESM3_ESM.tif]

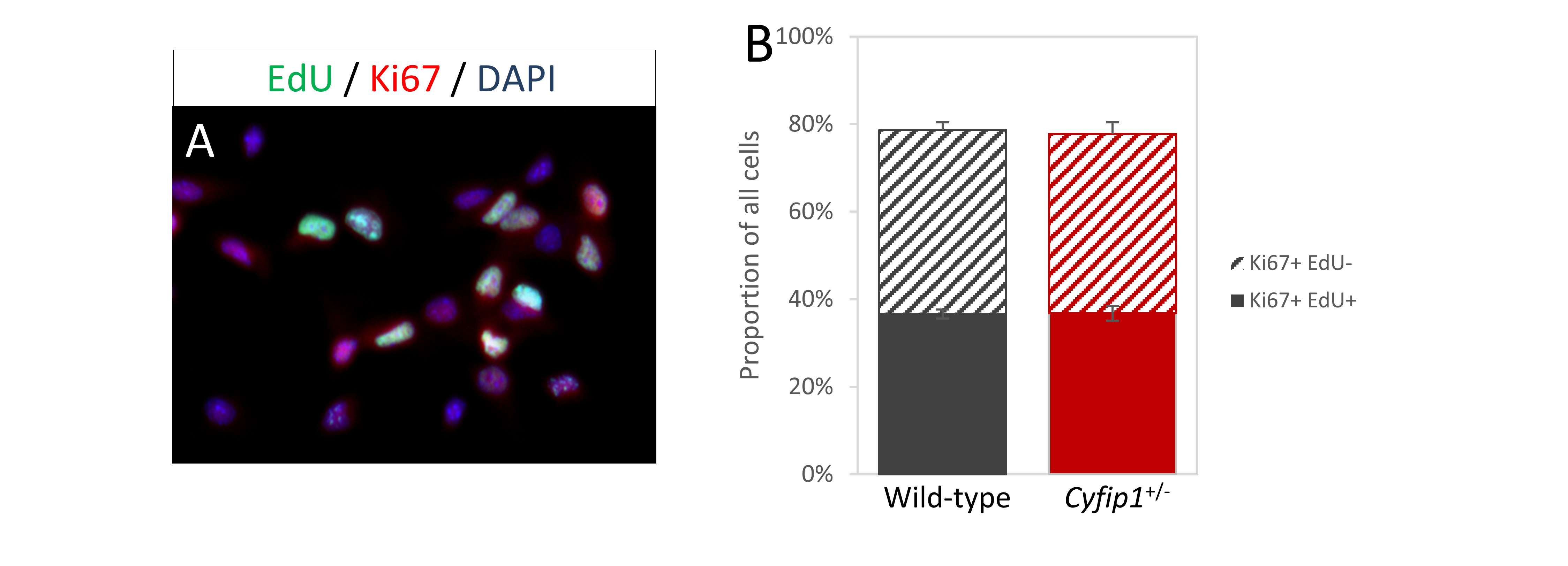

Supplement: Supplementary file 4 — Supplementary figur S3 [file 41398_2021_1415_MOESM4_ESM.tif]

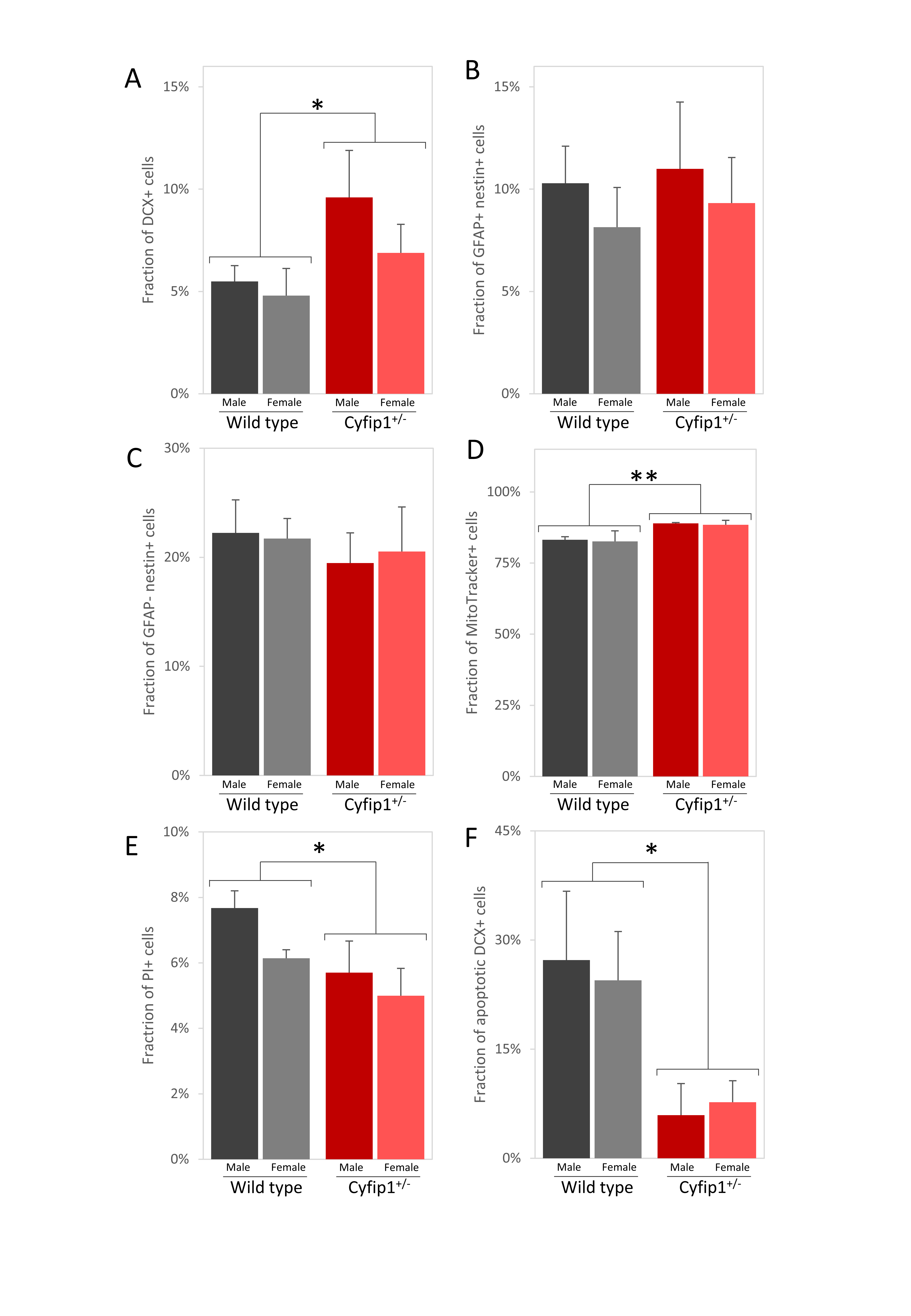

Supplement: Supplementary file 5 — Supplementary figur S4 [file 41398_2021_1415_MOESM5_ESM.tif]

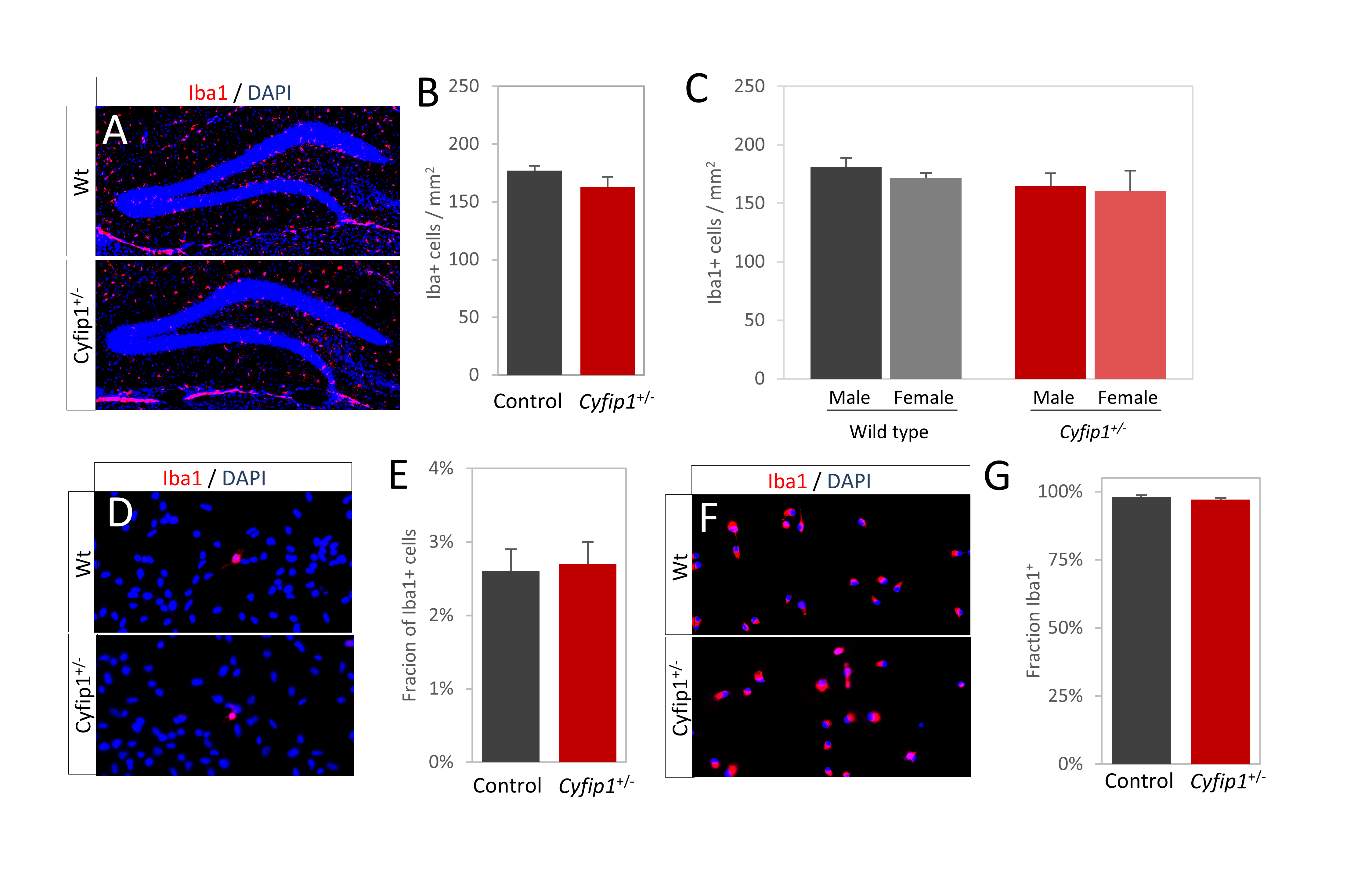

Supplement: Supplementary file 6 — Supplementary figur S5 [file 41398_2021_1415_MOESM6_ESM.tif]

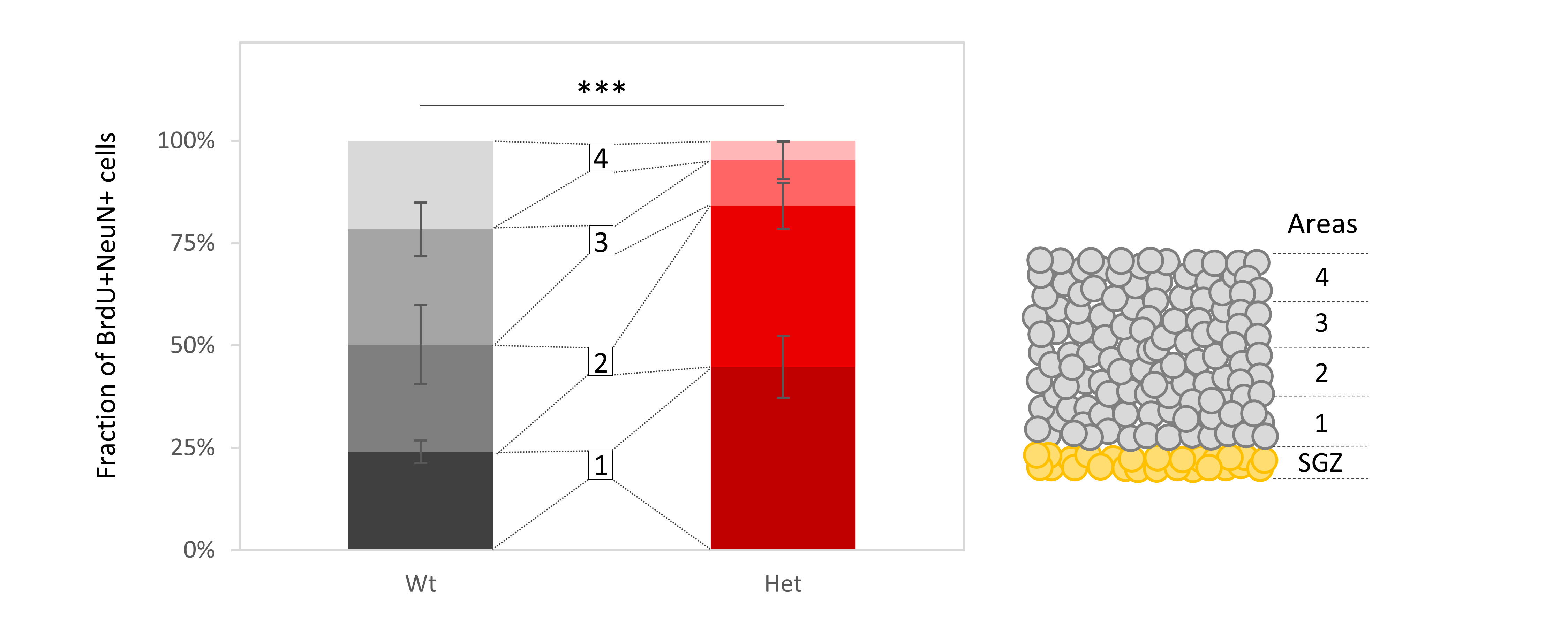

Supplement: Supplementary file 7 — Supplementary figur S6 [file 41398_2021_1415_MOESM7_ESM.tif]

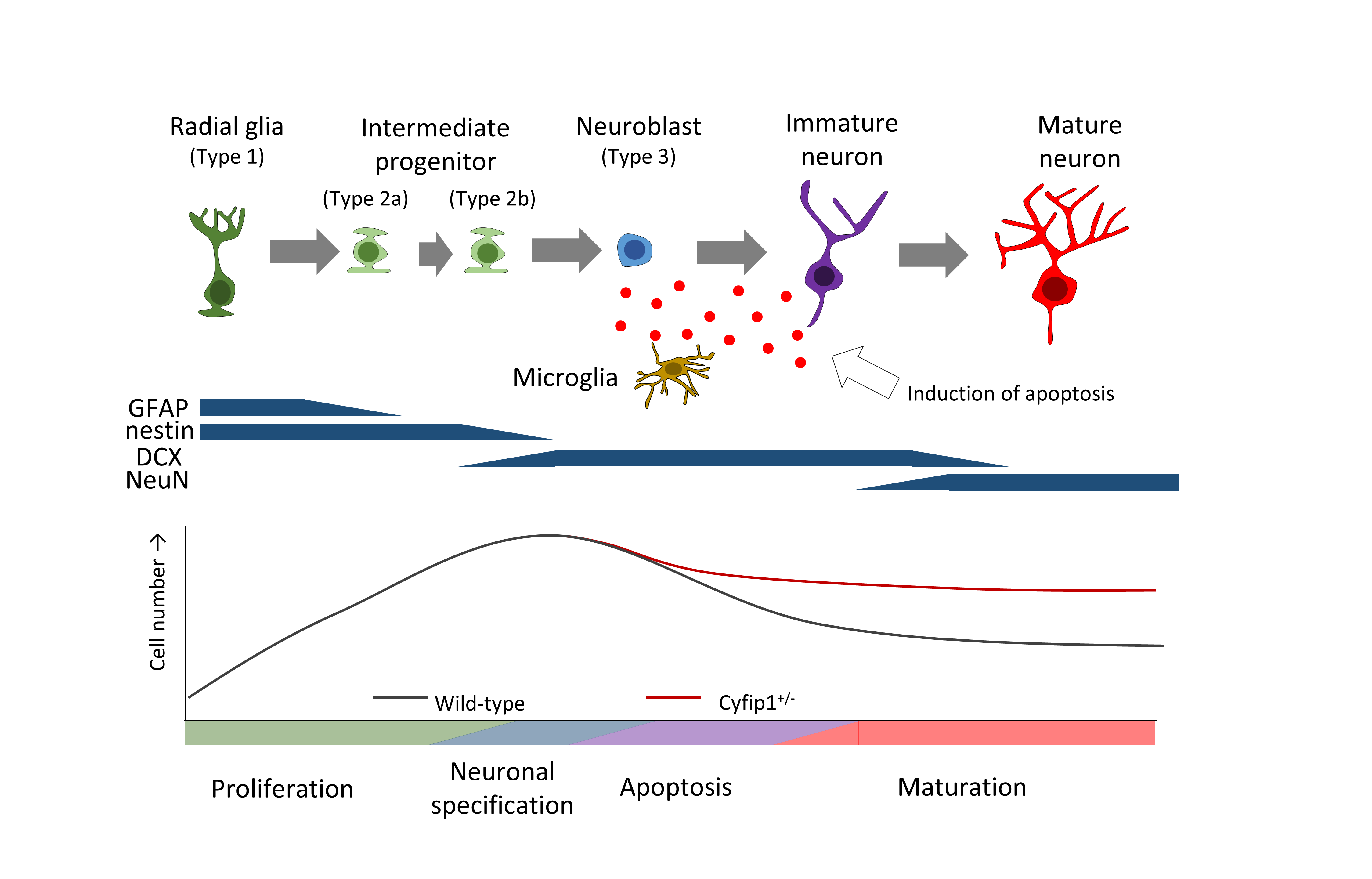

Supplement: Supplementary file 8 — Supplementary figur S7 [file 41398_2021_1415_MOESM8_ESM.tif]
